# Narcotics

**Published:** 1862-10

**Authors:** Joseph Bates


### NARCOTICS.

By JOSEPH BATES, M.D.

### ATROPA BELLADONNA.

*Whooping-Cough.*—Dr. Stille remarks "although previously employed in the treatment of this disease, belladonna was first systematically used to cure wooping-cough, by Schæffer and Wetzler, in 1810; the latter treated thirty children, all of whom were cured, between the eighth and fifteenth day after beginning to take the medicine. He found the most favorable period of the disease for commencing the treatment was between the sixteenth and twentieth days, and the proper dose to be a quarter of a grain of the root, morning and evening, for a child under one year of age. These results were confirmed by many writers, and particularly by Meglin, and by Kahleiss, in 1827. Several years later, Dr. Samuel Jackson, then of Northumberland, Pa., prescribed the remedy in numerous cases of whooping-cough, and with complete success, directing fractional parts of a grain of the extract at intervals of three hours, until dilatation of the pupil took place, when the medicine was ommitted altogether, or given in smaller doses. This treatment was preceded by antiphlogistic measures. Constant, in presenting a summary of his observations, at the Children's Hospital, in Paris, in regard to the treatment of whooping-cough, gives preference to belladonna over all other means; and his report shows substantial ground for this preference, inasmuch as the progress and decline of the disease corresponded with the suspension and resumption of the remedy. Dr. Thompson, of London, bears a similar testimony upon this subject. M. Fuster, Professor in the University of Montpellier, employed vapors of an infusion of belladonna leaves by inhalation, and with uniform success." Dr. Corson, of Norristown, Pa., says: "During the last seventeen years, I have given the extract of belladonna to hundreds of patients, from ten months to fifty years of age, and am firmly convinced that it has a greater control over whooping-cough than any other remedy in common use." Many observers have found it almost inefficacious, (says Stille) at the commencement of an epidemic, but very successful during its decline. So Ingmann states, that under such circumstances, it far surpasses all other medicines. M. M. Rilliet and Barthez do not appear to have used it, but call attention to the fact that Wendt and Gœlis have both seen mischievous consequences result from its use. Stille says: "No other writers allude to such effects, except as following an injudicious exhibition of the medicine, or the omission to suspend it when dilatation of the pupil comes on; even these disquieting symptoms are stated to have been temporary."

Bretonneau advised to be administered to a child of five years old, immediately before breakfast for two days, a pill containing one-twelfth of a grain of the extract; if no effect is observed by the third day, he raises the dose to one-eighth of a grain. He advises on the fifth day the same dose, and on the eighth day, if no effects are produced, one-third of a grain may be given, and continued for three days, or gradually increased until the pupils are effected, or the cough declines; at that point the dose should, for a time, remain the same. M. Trousseau remarks that insomnia is apt to be produced by belladonna in this disease, and he usually gives opium in combination with it. Cases of whooping-cough are reported, which were distinctly palliated when this drug was taken, and aggravated when withheld. On the whole, says Dr. Stille, it would appear that belladonna is positively curative in whooping-cough which has passed the inflammatory stage, but that in certain epidemics, it has signally failed, in the hands even of those who have before prescribed it with success. "The practitioner should be careful to watch its effects, lest they become alarming to the patient's family, or inflict even temporary mischief."

Belladonna, applied to a blistered surface, has been promptly and completely efficient in some cases of laryngitis, which were more or less of a spasmodic character.

*Braithwaite's Retrospect*, Part 42, p. 98, contains an article on the successful treatment of whooping-cough, by increasing doses of sulphate of zinc and extract of belladonna, by Dr. Fuller, physician to St. George's Hospital.—[In the *Medico-Chirurgical Transactions*, Dr. Fuller publishes a paper on the tolerance of belladonna by children. The following abstract of a paper read before the Harveian Society of London, contains a sufficient epitome of that article.] Dr. Fuller commenced his paper by calling attention to the commonly received opinion, as stated in most books on the subject, that, "Whooping-cough must run a certain course." He combatted this opinion by reference to the results of his own experience, and then proceeded to point out the causes which had led to its general adoption. Among these he mentioned the inefficiency of the treatment ordinarily employed, and the neglect of all measures likely to subdue the tendency to spasm on which the continuance of the whoop depends. Dr. Fuller regarded the complaint as consisting essentially of bronchitic irritation, usually not very severe, accompanied by reflex spasm of the air-passages, and expressed his opinion that in most cases the spasmodic symptoms are those which demand our most serious attention. Not only is the spasm a most painful symptom, but is one which may give rise to life-long mischief. His object, therefore, had always been to subdue the spasm as speedily as possible, and the practice which, until the last twelve months, he had found most successful for the purpose, was the administration of sulphate of zinc in rapidly increasing doses. (This Part was published in 1861.)

Dr. Fuller then referred to the discovery he had made as to the tolerance of belladonna by children, which is recorded in a paper published in the *Medico-Chirurgical Transactions*, and he briefly stated the facts which are there detailed in *extenso*, as to the amount of belladonna which may be given with safety, and the conditions which should be observed in its administration. The conditions he specified were, first, the remedy should be given at least four times daily, and should be administered at first in small doses, which may be increased day by day, or on alternate days, by a corresponding amount. He pointed out that mere dilatation of the pupils need not be regarded as a bar to its administration, and stated that if the precautions just referred to are observed, the daily dose of the extract of belladonna may be safely increased up to a scruple, or half a dram, without the production of any unpleasant symptoms. He then proceeded to state that he had brought these facts to bear on the treatment of whooping-cough, and from the conjoint use of sulphate of zinc and extract of belladonna, in rapidly increasing doses, had obtained results exceeding his most sanguine expectations. Rarely had he found the whoop to last above twenty-one days, and in some instances it had subsided in ten days. The mode in which Dr. F. proceeds, is to give the zinc and belladonna as soon as the whoop declares itself. If the attack is accompanied by much febrile excitement, and bronchitis, and irritation, he prescribes a cough-drop containing a dram of antimonial wine and a dram of ipecacuanha wine to two ounces of water, and, if necessary, applies a blister to the chest. Of the cough-drop a larger or smaller amount is given, according to circumstances. To children under three years of age, he usually begins by giving one-sixth of a grain of belladonna, and half a grain of sulphate of zinc, four times daily; and to children above that age one-quarter of a grain of extract of belladonna and a grain of sulphate of zinc. These remedies are given in a solution in water, and the dose of each substance is increased by a corresponding dose daily, or on alternate days; so that the child by taking one-quarter of a grain of the extract and one grain of the zinc, at a dose, would be taking one grain of the extract and four grains of the zinc at a dose, either on the fourth, the sixth, or the eighth day, according to the rapidity with which the dose is increased. Dr. F. concluded by citing cases illustrative of the value of this treatment; assured the members of its safety, and urged its general adoption.

In *Braithwaite's Retrospect*, Part 21, p. 141, M. Debregne has a paper on this subject. He says, "belladonna has been eminently useful in the epidemics of whooping-cough which he has observed; but the success attending its administration depends on the observance of the following rules. The dose of belladonna should be proportioned to the number of months representing the child's age; and the quantity to be taken in twelve days, (the ordinary duration of treatment,) will be five centigrammes (three-fourths of an English grain) multiplied by the number of months. Thus, for an infant six months old, the dose will be thirty centigrammes (four and a-half grains) in twelve days; for one of two years and a-half, the dose will be one and a-half gramme (twenty-three grains) in the same period. For children above six years of age, the quantity of three grammes (forty-six and one-third grains) is not exceeded. The medicine is always given three times in the day. For instance, the prescription for a child three years old would be powder of the root of belladonna, two grammes; to be divided equally into twelve powders, of which one is to be given daily, in three divided doses. If there be vomiting, it should be given immediately after a fit of vomiting and coughing. Recourse should not be had to this remedy, until the inflammatory element has been overcome by leeches, emetics, etc.; in other words, it is not to be employed before the tenth or fifteenth day, when the cough will have assumed its specific character.

*Dysmenorrhœa.*—Dr. Golden Bird found this agent very efficacious when there was no organic disease of the uterus, but shreds were discharged at the menstrual period, and severe pain was experienced at the same time in the hypogastric region. When the patient is anæmic or leucophlegmatic, one-fourth of a grain of extract of belladonna with one grain of sulphate of zinc may be given every two or three hours until the pain ceases. For plethoric patients ipecacuanha is more suitable than the sulphate of zinc. Between the catamenial epochs, purgatives should be administered, and other means taken to improve the general health. Bretonneau employed this remedy topically under similar circumstances. Dr. G. Bird's paper on dysmenorrhœa treated with belladonna was published in the *London Medical Times*, also in the *Boston Med. and Surg. Journal*, Vol. 30, p. 282. Dr. B. says in cases where the pain is seated over the region of the uterus, in the lower part of the abdomen, he has given the belladonna internally, taking care that the extract is properly prepared, and has invariably found it successful; great relief is experienced at the next menstrual period; greater still at the succeeding one, and a cure is finally accomplished. By removing (says Dr. Bird,) the irritable condition of the uterus, and curing the dysmenorrhœa, the pseudo-pleurisies and pseudo-peritonitis, for which bleedings were formerly, unfortunately, so largely practiced, will soon get well. The cases in which the pain is seated chiefly in the loins, do not yield to the employment of belladonna. This, Dr. Bird considered was owing to congestion of the uterus. Fortunately (he says) the cases that are most remarkable are the most frequent.

Dr. Walker (as stated in the *Lancet*, March 23, 1844, p. 27,) has found belladonna alone, both externally and internally, in cases of dysmenorrhœa to afford temporary relief. He applies it externally in the form of a plaster of the simple extract spread on adhesive plaster.

He has seen the belladonna in conjunction with hydrocyanic acid, in the proportion of one-quarter to half a grain of the extract to three minims of Scheele's preparation, four or five times a day, of great benefit in cases of severe gastrodynia.

*Constipation.*—Trousseau declares belladonna to be the remedy par excellence for habitual constipation. It does not purge nor produce loose stools, but only renders defecation easier, and sometimes in the dose of a quarter of a grain the extract will produce several stools. As soon as the bowels become regular the dose of the medicine should be gradually diminished Cases illustrative of the efficacy of this treatment are reported by Fiessenger, who, however, made use of suppositories containing the extract of belladonna, by Blacke, and by Flewry. (Stille).

Copland recommends small doses of belladonna in alvine obstructions (p. 477).

*Strangulated Hernia.*—In truly spasmodic affections, or those in which there is abnormal, and, for the most part, paroxysmal muscular contraction, belladonna has been extensively and beneficially employed. David reports two cases in which the internal use of belladonna led to the reduction of strangulated hernia, which seemed to demand an operation. He gave half a grain of the extract every half hour. In one case three, and in the other four doses were taken.

In the *Gazette Hebdomaire*, there is reported a case of inguinal hernia, which was relieved after taxis had failed, by the administration of the extract of belladonna, in three or four grain doses every half hour. The tincture of belladonna was also employed locally by means of a flax-seed poultice.

In the same journal there is also a report of a case in which belladonna was administered, with very great relief to the patient. In this there was no protrusion of the bowel, but, from the symptoms, obstinate constipation, great pain, stercoraceous vomiting, etc., it was evident that there was complete occlusion of the bowel. The belladonna in this case was employed after an operation for artificial anus had been decided upon. The remedy was used in the form of an ointment, by friction; and as soon as complete intoxication was induced its good effects were preceptible.

*American Journal*, Vol. 15th, p., 533: Dr. Frankel has successfully treated six cases of strangulated hernia, with the extract of belladonna. Five of these cases were crural hernia in females. The sixth was an umbilical hernia.

The author was called upon to visit a boy some ten years of age, July 8th, 1862; found the little patient in great distress; and upon examination of the abdomen readily discovered the cause of his distress, which was inguinal hernia. I made every endeavor to reduce it, but failed in accomplishing it. I then made an ointment of belladonna and lard, equal parts, and spread a small plaster, and applied it to the hernial tumor, and waited its results. In less than half an hour my patient became quiet and easy; and his pupils some dilated. I then directed my attention to the hernia, and found a spontaneous reduction. The plaster was immediately removed, and the surface cleansed with tepid water; nevertheless the pupils remained dilated for twenty-four hours.

*Rigidity of the os uteri during labor.*—It was first recommended to overcome this condition by Chaussier. He as well as several other writers have testified to its virtues in overcoming this annoying incident of parturition.

This condition of the os uteri may readily be controlled by an ointment made of belladonna and lard, and with the finger apply it immediately to the mouth of the uterus.

In the *American Med. Monthly*, may be found a paper of B. F. Barker's on belladonna shortening labor. Dr. Barker gives a table of 147 cases of labor, in which this remedy had been used for dilating the os externum by comparatively painless contractions. The extract was administered in quarter-grain doses, two or three times daily, commencing about two weeks previous to the close of gestation.

Plethoric patients took tartar emetic with belladonna—three grains of the former, eight of the latter, in two ounces of syrup of orange peel (one ounce of the tincture of orange peel, and one ounce of water), a teaspoonful three times a day. With some the following formula was used: compound tincture of cinchona, three ounces; syrup, one ounce; ext. of belladonna, eight grains. Other combinations for special indications. A very great difference appeared in the susceptibility of patients to the influence of the agent, owing to the difference in the purity and strength of the article. One would seem to have double the potency of another, without any corresponding difference in the appearance, color, or odor. In some cases the dose had to be diminished, but in most instances it could be gradually doubled, or even tripled. Dryness of the throat, slight uneasiness or giddiness of the head, dimness of the vision, are indications to diminish the dose. Not one of the children was still-born, and in none of the cases was there post-partum hæmorrhage or retention of the placenta. In one the function of lactation was entirely absent; in two others the mammary secretion did not appear until the fifth day.

The following cases are mentioned in the *Boston Medical and Surgical Journal*, Vol. 30, p. 502:—"Premature delivery. Six and a-half months gone. Called twelve hours after birth of fœtus, to deliver placenta. Placenta adherent. Uterus collapsed upon the same. Could not dilate the os tincæ with my finger. Used the ungt. belladonna to the os and neck; in thirty minutes the uterus was dilatable. Delivered with the blunt hook. Patient had a quick recovery."

Another:—"Protracted labor; rigidity of the os tincæ; alarming hiccough and vomiting, with sudden cessation of expulsive pains. The slightest touch of the finger to the os, or pressure of the child from change of position, would induce the hiccough and increase the vomiting. Applied the ungt. to the os tincæ. In five minutes the hiccough ceased, the vomiting soon followed, the rigidity relaxed, and the patient fell into a quiet sleep. With the aid of savin and ergot, in an hour and a-half the patient was delivered."

The author, R. P. Stevens, closes by stating that in his materia medica there is not a more potent drug than the atropa belladonna.

*Laryngitis.*—Dr. Stille says that belladonna applied to a blistered surface, appears to have been promptly and completely efficient in some cases of laryngitis which displayed more or less of a spasmodic element; and as an internal remedy for nervous coughs, especially for those which, independently of any local disease of the respiratory apparatus, disturb the rest at night by their persistence and violence.

Richard Hughes (Surgeon to the Brighton Orthopædic Hospital,) has a publication on the influence of belladonna on the pneumogastric nerve, in *Braithwaite*, part 42, p. 296. He closes his able communication as follows:—"The practical results from the above facts will be, that we shall be led rationally to the use of belladonna and its congeners in all affections of the pneumogastric nerve. Laryngismus stridulus must, therefore, be added to the above mentioned morbid conditions. I have lately used belladonna (in combination with nitric acid) in every case of common cough which has come under my notice, and with far more marked success than I have obtained from any other remedy. I trust that a more extended trial may add fresh support to the view thus advocated."

*Tetanus.*—Dr. Hutchinson has reported many cases of traumatic tetanus, as cured by the extract of belladonna. Dr. H. reports that the specific action of the medicine upon the pupils was followed by an abatement of the spasms, but this was not until the dose was increased from half a grain or a grain of the extract every three hours, to four grains every two hours. It was then gradually diminished. The treatment continued about one month.—(*Am. Jour. of Med. Sci.*, Apr. 1858, p. 340.)

Vial mentions three cases of cure by this agent, and Ernest reports others. Belladonna is said to have cured idiopathic form of tetanus. The following case is taken from the *Gazette Medicale;*—published also in *Braithwaite's Retrospect*, Part 22, p. 90.

"M. Bresse, surgeon at the Military Hospital at Rennes, proposed, in 1848, the treatment of traumatic tetanus by the application of tincture of belladonna, and reported a case, in which it had been successfully employed in the *Gazette Medicale de Paris*, of Sept. 30, 1848. M. Bresse has now placed on record another case, which has come under his notice. The patient, one of the Garde Mobile, received a wound on the 20th of March; tetanic symptoms appeared on the 5th of April. Frictions of belladonna were commenced on the 5th, and by the 12th the patient was out of danger. Imprudently exposing himself to cold, the tetanic symptoms returned in the muscles of the back, but were quickly removed by again having recourse to frictions. The tincture employed was composed of five parts of extract to eleven of alcohol, and was applied all over the body, and more particularly over the rigid parts. M. Bresse adds, that another practitioner has arrested trismus, which he feared would proceed to general tetanus, by the same means."

From the same work, Part 39, p. 315, a short notice is taken from *Med. Times and Gazette*, April, 1854: "Belladonna may be given in large doses, in tetanus. Give one grain every two hours, and increase this dose to one and a-half grains, using occasionally a suppository with three grains in it. Watch the pupil; if it dilate, gradually desist from the belladonna.

From the same work, Part 10, p. 29: "In tetanus larger doses of belladonna are endured and required for its relief than in cases of neuralgia. The enormous dose of five grains of the extract of belladonna was successfully administered in one case, and in another the dose was gradually increased, until eventually four grains were given, and repeated every two hours. In this case, after a certain quantity of the medicine had been administered for a certain time, the system appeared to acquire a tolerance of it, and, from this point of improvement the symptoms remained stationary until an increased quantity was administered, when speedily a manifest advancement again took place, and this continued for a certain period, until a larger dose was again demanded, in order still further to influence the disorder. This course was very well marked and positive, and this it was which encouraged and induced Dr. H. still further to increase the dose of belladonna, until eventually the tetanus was entirely subdued."—*Journal of Materia Medica.*

